# TGF-β Superfamily Signaling in the Eye: Implications for Ocular Pathologies

**DOI:** 10.3390/cells11152336

**Published:** 2022-07-29

**Authors:** Soumaya Hachana, Bruno Larrivée

**Affiliations:** 1 Maisonneuve-Rosemont Hospital Research Center, Montreal, QC H1T 2M4, Canada; soumaya.hachana@umontreal.ca; 2 Department of Ophthalmology, Université de Montréal, Montreal, QC H3C 3J7, Canada

**Keywords:** TGF-β, BMP, ocular diseases, age-related macular degeneration

## Abstract

The TGF-β signaling pathway plays a crucial role in several key aspects of development and tissue homeostasis. TGF-β ligands and their mediators have been shown to be important regulators of ocular physiology and their dysregulation has been described in several eye pathologies. TGF-β signaling participates in regulating several key developmental processes in the eye, including angiogenesis and neurogenesis. Inadequate TGF-β signaling has been associated with defective angiogenesis, vascular barrier function, unfavorable inflammatory responses, and tissue fibrosis. In addition, experimental models of corneal neovascularization, diabetic retinopathy, proliferative vitreoretinopathy, glaucoma, or corneal injury suggest that aberrant TGF-β signaling may contribute to the pathological features of these conditions, showing the potential of modulating TGF-β signaling to treat eye diseases. This review highlights the key roles of TGF-β family members in ocular physiology and in eye diseases, and reviews approaches targeting the TGF-β signaling as potential treatment options.

## 1. Introduction

Pathologies involving angiogenesis are particularly devastating in terms of visual function. Among these, age-related macular degeneration (AMD) is one of the main causes of blindness in developed countries, especially in people over 50 years of age. Its prevalence is likely to increase to 288 million people by 2040 [1,2]. In the North American population, it is estimated that 15 million people (85% of all patients with AMD) have the dry form and 1.7 million (15% of patients with AMD) have the wet form (with neovascularization) [3]. In the process leading to the formation of choroidal neovascularization (CNV), neovascular growth is coupled with focal proteolytic degradation of the Bruch’s membrane.

A better understanding of the CNV mechanisms has led to the development of new therapeutics focusing on VEGF signaling inhibition, such as Lucentis (Ranibizumab), Eylea (Aflibercept), and bevacizumab (Avastin) [4]. Although these treatments improve visual acuity, decrease vascular permeability, and prevent neovascularization in patients with wet AMD, repeated intra-vitreous injections increase the risk of infection, cataracts, glaucoma, increased intra ocular pressure, retinal detachment, endophthalmitis, and systemic toxicity [5,6]. In addition, the administration of VEGF inhibitors could be ineffective in 7–15% of ocular neovascular patients [7], which represents a major drawback of these treatments. Thus, there is a need for alternative strategies, either as monotherapies or which could be associated with anti-VEGF treatments to achieve combination therapy more likely to produce a greater therapeutic benefit for wet AMD patients.

Members of the Transforming Growth Factor-β (TGF-β) superfamily have been implicated in several ocular pathologies, including wet AMD, corneal inflammation, and fibrosis following tissue injury or primary open-angle glaucoma. The TGF-β superfamily is composed of a group of cytokines, including TGF-β, Bone Morphogenetic Proteins (BMP), Growth Differentiation Factors (GDF), and Activins, that play important roles in embryonic development, tumorigenesis, and inflammatory responses [8]. Depending on the cellular context, the TGF-β family members can either inhibit or stimulate proliferation, control the turnover of the extracellular matrix, as well as be involved in epithelial–mesenchymal interactions during embryogenesis. Their activities are also involved in tissue repair and the modulation of the immune response. The deregulation of TGF-β signaling induces abnormalities during embryonic development [9] and has been implicated in several human pathologies, such as cancer [10], tissue fibrosis [11], or autoimmune diseases [12]. In recent years, the role of TGF-β family members in neovascularization has gradually been revealed. It has been found that the expression of several TGF-β family members is significantly altered in AMD patients [13] and in experimental models of laser-induced CNV [14]. TGF-β signaling plays a central role in promoting CNV by regulating VEGF production and signaling, immunity, inflammation, and vascular fibrosis [14]. In the present review, we examine the complex roles of TGF-β family members and their signaling pathways in the pathogenesis of ocular diseases. 

## 2. The TGF-β Superfamily

### 2.1. Soluble Factors of the TGF-β Superfamily 

The TGF-β cytokines were first discovered in the early 1980s and three TGF-β isoforms have been identified in mammals (TGF-β1, TGF-β2, and TGF-β3) [15,16]. Since then, over 40 proteins of the TGF-β superfamily have been uncovered and classified into different subfamilies, including TGF-βs, activins and inhibins, BMPs, GDFs, anti-Müllerian hormone (AMH), and Nodal [8,17]. The TGF-β family ligands exert their biological activities as homodimers or heterodimers, which are covalently linked by disulfide bonds.

Activins and inhibins, comprised of activin A, B, AB, C, and E as well as inhibin A and B isoforms, participate in a variety of biological processes and have been implicated in the early stages of embryonic development and the function of many human tissues and organs, and regulate several biological processes, including cellular proliferation, differentiation, and invasivenes [18,19]. Activins and inhibins share common β subunits, with activins being composed of ββ homodimers while inhibins are composed of αβ heterodimers. For activins, the dimerization of the two β-subunits—termed inhibins βA or βB—gives rise to the formation of activin A (βAβA), activin B (βBβB), and activin AB (βAβB). Inhibins are composed of heterodimers consisting of a subunit called inhibin α and either one of the two β-subunits to give rise to inhibin A (αβA) and inhibin B (αβB) [20,21].

In addition to the classical β subunits, two further mammalian subunits, termed activin βC and βE, have also been described. While few biological roles for either subunit have been elucidated, they have been proposed to play a role in hepatic regeneration [22,23]. So far, their expression has not been observed in the eye and they have not been reported to be implicated in ocular pathologies.

The family of BMPs, whose members are particularly known for their roles in osteogenesis and neurogenesis, are composed of BMP2-15, GDF1-3, GDF5-11, GDF15, and Nodal (BMP16 in zebrafish). Using transgenic animals, more insights into the functions of BMPs have been gained and these reflect their importance in several developmental processes. BMP2, 6, and 9 play important roles in the induction of mesenchymal cell differentiation into osteoblasts [24]. BMP7 has been shown to contribute to eye and limb development [25]. BMP4, 7, and 14 are required for reproductive tissue development [26]. BMP2, 3, and 7 are involved in cartilage regeneration while BMP12 and 13 are important for normal tendon healing [24,27,28]. Nodal also plays an essential role in the early stages of development and mesoderm formation [29].

Other soluble factors of the TGF-β superfamily also have diverse roles during development. The Müllerian Inhibitory Factor (MIF) subfamily consists exclusively of MIF, also known as Anti-Müllerian Hormone or Müllerian Inhibitory Substance, and plays a critical role in sexual differentiation [30].

Proteins from the TGF-β superfamily display several structural and functional similarities. Indeed, most of them are synthesized in the form of a pre-proprotein dimer. A cleavage is carried out by proteases of the Subtilisin-like Proprotein Convertase family to obtain a mature form which is then secreted in the extracellular medium [31]. There are exceptions, such as the precursor of Nodal, which is able, under certain conditions, to attach to its receptor and to induce signaling without having undergone maturation [32]. In addition, certain proteins of this family require additional modifications. This is the case of TGF-βs and certain GDFs (8 and 11) which are secreted in a non-active form and will need the action of a metalloprotease in order to release the fully active form [33].

### 2.2. Receptors and Their Signaling

The ligands of the TGF-β family bind to two types of transmembrane receptors (types I and II). These receptors share structural similarities and are composed of an extracellular domain rich in cysteines, a transmembrane domain, and a highly conserved intracellular domain exhibiting serine threonine kinase activity (Ser-Thr) [34]. There are seven type I receptors, also called ALK (activin receptor-like kinase), as well as five type II receptors. Type I receptors are structurally distinguished from type II receptors by the presence of a glycine-rich GS region. The latter plays an important role in the signal regulation and transduction [35].

**Figure 1:**
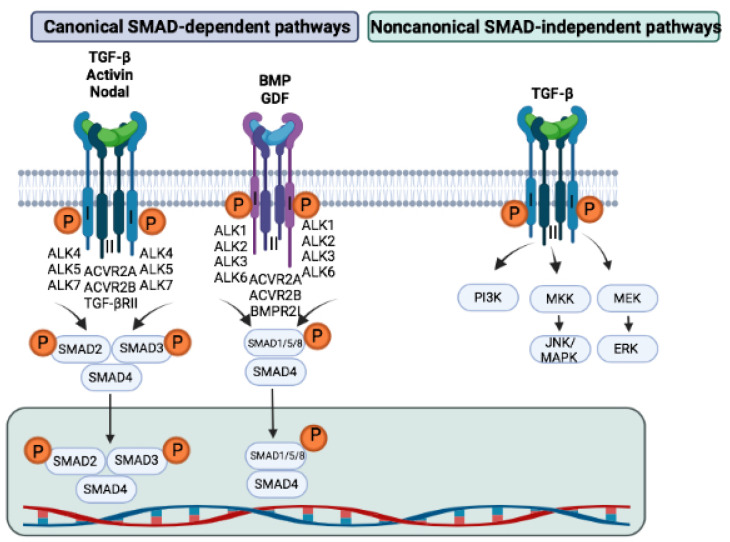
TGF-β superfamily signaling pathways. TGF-β superfamily proteins exert their cellular activity via canonical and noncanonical TGF-β signaling pathways. The activation of the heterotetrameric complex of TGF-β type I and type II receptors leads to phosphorylation and activation of SMAD proteins. In addition, TGF-β superfamily ligands can also activate noncanonical signaling pathways, such as the phosphatidylinositol 3-kinase (PI3K)/AKT, extracellular signal-regulated kinases (ERK), Jun N-terminal kinase family (JNK), and mitogen-activated protein kinase (MAPK) signaling pathways.

The signaling cascade is initiated when the ligand binds to a constitutively active dimer of type II receptors (Figure 1). This liaison allows the recruitment of a dimer of type I receptors and the formation of a heterotetramer. The type I and II receptors do not interact directly with each other, but via ligand binding. The type II receptor phosphorylates the type I receptors at the level of serines and threonines of the GS domain. In the absence of ligand binding, the GS domain serves as a binding site for FKBP12, a pathway inhibitor [31]. The phosphorylation of the type I receptor allows the detachment of this inhibitory protein, making the GS domain available and then serving as an attachment site for cytoplasmic proteins, such as SMADs. Regulatory (R-) SMADs are then phosphorylated and can play an important role in the regulation of transcription [35].

### 2.3. SMADs

The intracellular signaling of the TGF-β family is regulated by SMADs. There are three types of SMADs: Regulatory (R-) SMADs (SMAD1, SMAD2, SMAD3, SMAD5, and SMAD8/9), Common mediator (Co-) SMADs (SMAD4) and Inhibitory (I-) SMADs (SMAD6 and SMAD7).

Phosphorylation of the type I receptor allows the recruitment of Regulatory SMADs and destabilizes the interaction with the SARA (SMAD Anchor for Receptor Activation) protein. SMAD2 and SMAD3 can be specifically immobilized near the cell surface SARA, via the interactions between a peptide sequence of SARA and an extended hydrophobic surface area on SMAD2/SMAD3 [36]. SMADs are then phosphorylated, at the level of two serine residues, which allows them to form homomeric and heteromeric complexes and to interact with SMAD4 [37]. These SMADs complexes accumulate in the nucleus and regulate the expression of their target genes [38]. The regulatory SMADs and SMAD4 both have two highly conserved globular domains, MH1 and MH2. The MH1 and MH2 domains, located in the N- and C-terminal positions, respectively, are linked by a variable intermediate region rich in proline [39]. Each combination of SMADs with a different partner targets a number of specific genes depending on the specificity of the DNA binding of that combination. For example, among the genes regulated by this family, several play critical roles in vascular development, such as VEGF, Plasminogen Activator Inhibitor-1, ID1, ID3, or uPAR. Studies in transgenic mice have also shown that SMAD3 and SMAD4 were essential for the induction of epithelial to mesenchymal transition (EMT) by TGF-β [40]. TGF-β and SMAD proteins also regulate the induction of EMT through the modulation of transcription factors of the Snail family, the ZEB family, and the bHLH family, thus leading to down-regulation of epithelial markers and up-regulation of mesenchymal markers [40,41].

### 2.4. The Regulation of TGF-β Signaling

The activity of type I and II receptors of the TGF-β family can be regulated by a third class of receptors, the type III receptors betaglycan and endoglin. These are transmembrane proteins that have a large extracellular domain and a short intracellular domain lacking kinase activity [42]. Betaglycan has a strong affinity for TGF-β1, 2, and 3, for activin A, as well as for BMP2, 4, 7, and GDF15 and increases the affinity of its ligands for their type I and type II receptors [43]. While it is not involved directly in TGF-β signal transduction, betaglycan also modulates TGF-β by acting as a reservoir of ligand for TGF-β receptors [44]. Endoglin is a receptor preferentially expressed on the surface of endothelial cells and has a significant affinity for TGF-β1, activin A, BMP2, and BMP7 [45]. In addition, endoglin is able to bind BMP9 and TGF-β3 without the intervention of other type I or II receptors [45]. Endoglin may participate in the angiogenic response by modulating the response of ALK1 to blood flow shear stress [46].

Several soluble factors also regulate the activity of soluble ligands through their interactions with these factors. In the extracellular space, the activity of BMPs is regulated by the secreted factors Chordin and Noggin, which interact with BMP proteins and interfere with binding to their receptors [47,48].

SMAD signaling is also tightly regulated and involves negative control loops controlled via the Inhibitor SMADs, SMAD6, and SMAD7. SMAD7 recruits two E3 ubiquitin ligases and presents them to the receptor. This allows the initialization of its degradation and the termination of the signal [17]. As Inhibitor SMADs have an MH2 domain allowing them to interact with receptors, they are also able to bind to Regulatory SMADs and Cooperating SMADs. Consequently, ubiquitin ligases and phosphatases can also stop the signaling at the level of these two types of SMADs [17].

Furthermore, the role of heat shock protein 90 (HSP90) in the regulation of TGF-β signaling has been highlighted. HSP90 is a molecular chaperone referred to as the cancer chaperone due to its large cohort of oncogenic proteins [49]. HSP90 has been shown to enhance TGF-β1 pathway activation in Müller Glial cells [50] and regulates TGF-β signaling by binding to TGF-βRI and TGF-βRII, preventing their degradation in non-malignant human cells [49]. Over-expression of HSP90 considerably alters the signal transduction pathway mediated by TGF-β1. Indeed, Tosi et al. show that increased expression of HSP90 results in over-expression of TGF-βRII+, SMAD2, and SMAD3 [50]. In addition, it has been reported that HSP90 negatively regulate TGF-β in the osteosarcoma cells in vitro by binding to the latency-associated peptide (LAP) of inactive TGF-β [51]. De la Mare et al. described the effect of a synergy between extracellular TGF-β1 and Hsp90β on downstream signaling in colon cancer cells. It was found that TGF-β1 and Hsp90β work synergistically in mediating adhesion and migration [52].

## 3. Biological Roles of TGF-β

The TGF-β pathway is involved in a large number of physiological phenomena, including morphogenesis, embryonic development, and inflammation. Signaling dysfunction of several of its members is associated with pathologies such as cancers or cardiovascular diseases.

TGF-β family members exert a significant antiproliferative effect on epithelial, endothelial, hematopoietic, neural cells, and in certain types of mesenchymal cells [42]. The growth arrest induced by TGF-β family involves different mechanisms, such as the transcriptional induction of cyclin-dependent kinase inhibitors, p21^WAF1^ and/or p15^INK4B^, the inhibition of c-Myc, or Cdc25A phosphatase expression [53]. TGF-β also regulates the expression of extracellular matrix proteins (ECM), including fibrillar collagen and fibronectin. Furthermore, it also suppresses degradation of ECM by inhibiting the expression of metalloproteinases and serine proteases, and by increasing the expression of protease inhibitors, such as Tissue Inhibitor of Metalloproteinases or Plasminogen Activator Inhibitor-1.

### 3.1. In Vascular Development

Impairment of signaling by several TGF-β family members leads to significant defects in the differentiation and maturation of the primary vascular network. Studies have demonstrated the importance of the TGF-β pathway, particularly the ligands TGF-β1, BMP9 and the receptors ALK1, ALK5, TGF-βR2, and endoglin, in developmental angiogenesis and in maintenance of the integrity of adult blood vessels [54,55]. The role of the TGF-β superfamily members in vascular morphogenesis has been highlighted using different knockout mice of TGF-β family signaling components [56]. This has allowed the demonstration that TGF-β1 participates to the maintenance of the integrity of blood-retinal barrier [57,58]. In addition to the induction of VEGF production, TGF-β1 also activates the expression of fibroblast growth factor and platelet-derived growth factor, potent angiogenic mediators in neovascularization [59,60,61]. Furthermore, it has been shown that TGF-β1 deficient mice die during midgestation partly due to defects in angiogenesis [62]. TGF-β1 signaling mediated by ALK1 has been reported to be essential for the transition of endothelial cells from the activation phase to the resolution phase of angiogenesis, whereas TGF-β1 signaling via ALK5 induces the secretion of proteinases and promotes the resolution phase of angiogenesis [54]. Together, these two type I receptors of TGF-β have been proposed to regulate the activation state of endothelial cells [54,63].

Mice lacking ALK1, its co-receptor endoglin, or the common effector SMAD4, are involved in the pathogenesis of hereditary hemorrhagic telangiectasia (HHT), characterized by the presence of telangiectasias and arteriovenous malformations [64,65]. Park et al. [66], showed that the appearance of hemorrhages and arteriovenous malformations following ALK1 inactivation leads to the death of these mice. This confirms the importance of continuous ALK1 signaling to maintain vascular homeostasis and vessel integrity [67]. ALK5 enhances the expression of the endothelial component of claudin-5 [68,69] tight junctions, and ALK5-null embryos exhibit defects in the formation of vascular smooth muscle layers and retinal blood barrier [70,71]. Endoglin, which modulates BMP/TGF-β signaling by interacting with TGF-βR1 and R2, is enriched in highly proliferating vascular endothelial cells [72], promotes TGF-β/ALK1 signal transduction, and indirectly limits TGF-β/ALK5 signaling [73]. As such, endoglin is a promising vascular target that can be used as a marker of tumor angiogenesis, and has been successfully used to target endothelial cells in anti-angiogenic therapies [74]. Postnatal conditional deletion of Endoglin or ALK1 in mice leads to development of arteriovenous malformations [75], and has been associated with the development of HHT [76].

Other secreted ligands of this family also have an impact on the formation of blood vessels and are known to participate in the regulation of angiogenesis. For instance, BMP7 inhibits the growth of vascular smooth muscle cell (SMC) and maintains the expression of vascular SMC phenotype [77]. BMP2 is essential for early embryonic development and it prevents platelet-derived growth factor-induced vascular SMC proliferation [78].

Mice lacking BMP2/4 are early embryonic lethal due to extraembryonic malformations and showed abnormal development of the vasculature. Indeed, BMP4 promotes the proliferation and migration of endothelial cells via the stimulation of VEGF-A and Tie-2 signaling [79]. BMP4 has a separate role from BMP2 about vascular morphogenesis. BMP4 is essential for endothelial branching while BMP2 is responsible for the inhibition of endothelial branching [80]. Furthermore, BMP6 was shown to induce endothelial cells migration and tube formation via SMAD1/5/8 phosphorylation [81]. Finally, among the different BMPs, BMP9 and BMP10 have a particularly important role in the physiology of endothelial cells [82,83]. This is supported by the fact that BMP9 and BMP10 bind with high affinity to ALK1 and endoglin, and their signaling leads to vascular quiescence [84,85].

Gene ablation studies underlined the importance of TGF-β receptors ALK5 and ALK1 for the regulation of angiogenesis in animal models. In endothelial cells, the balance between activation of ALK1 and ALK5 signaling pathways is crucial for vascular homeostasis. TGF-β/ALK5/SMAD2/3 and TGF-β/ALK1/SMAD1/5/8 signaling on angiogenesis suggest a scenario in which TGF-β could play opposite effects. According to this model, ALK1 would promote the process of endothelial cells migration and proliferation while ALK5 would inhibit it [63,75,86,87,88]. These completely opposite effects on angiogenesis could be due, among other factors, to the level expression of ALK1. For example, during embryonic life Alk1 is strongly expressed in vascular endothelium but its level expression decreases during adult life [89,90]. However, in response to angiogenic stimulation, the expression level of Alk1 increases [89,90]. The consequent altered balance between ALK1 and ALK5 pathways could mediate the angiogenic effects of TGF-β members [86]. In complex with ALK1, ALK5, and TGF-βRII, endoglin promotes proliferation of endothelial cells, where its expression level increases during angiogenesis [73]. On the other hand, Tual-Chalot et al. [91] showed that genetic depletion of endothelial Eng in adult mice leads to increased angiogenesis and arteriovenous malformations. Other studies demonstrate an antiangiogenic role of ALK1 during angiogenesis [88]. It has also been demonstrated in postnatal mice that ALK1 signaling inhibited angiogenesis via the Notch pathway [75].

### 3.2. In Immune Cells

TGF-β family members were shown to reduce the proliferation and affect the survival of T cells [92,93]. TGF-β ligands are also considered key cytokines in the differentiation of pro-inflammatory human Th17 cells, which have a crucial role in immunity and in the development of autoimmunity [94,95]. Furthermore, a direct link between Treg, responsible for the negative regulation of immunity [96], and TGF-β has been discovered. For instance, TGF-β1 promotes the induction of IL-10-secreting CD4(+) Treg cells through Foxp3 production, a regulator of Tregs in naïve T cells [97]. TGF-β1 is also known to play an important role in the immune system via the regulation of lymphocyte proliferation, differentiation, and survival [98]. It controls adaptive immunity by directly promoting the expansion of Treg cells and by inhibiting the function of effector T cells and antigen-presenting dendritic cells [95]. Otherwise, TGF-β family members can suppress adaptive immune responses via the function of Tregs and by suppressing Th1 cells, Th2 cells, and CD8+ T cells [99], but also enhance the adaptive responses through the induction of Th17 cells, Th9 cells, and CD4+ [95].

TGF-β1 has also a multitude of functions, including the control of the proliferation and the differentiation of several other cell types involved in the immune response. It regulates adhesion and migration in various cells, such as macrophages, activated B cells, immature hematopoietic cells, neutrophils, and dendritic cells [100]. TGF-β1 knockout mice eventually die from severe tissue necrosis and a generalized inflammatory response a few weeks after birth.

Humoral immunity is also affected by TGF-β signaling. Indeed, TGF-β had been shown to inhibit B cell maturation, proliferation, and IgM and IgG production [101]. An important mechanism at play to promote immunity is the production of IgA [102]. TGF-β is the major cytokine signal involved in the generation of IgA [102], which prevents the multiplication and systemic spread of pathogenic bacteria allowing the maintenance of the intestinal tissue barrier [103]. Coffman et al. demonstrated that IgA switching is induced by TGF-β, leading to increases in the amount of IgA secreted by the B cells [104].

Other studies have also described a pivotal role for BMPs regulating the differentiation of T cells. Most of these studies are based on mouse and cell line models [105,106]. BMP2 and BMP4 are typical representatives of BMPs that affect T cell development in the mouse thymus [107]. Indeed, the BMP pathway is critical for human naive CD4+ T cell activation and homeostasis. Through the stimulation via T cell receptor, human CD4+ T cells are able to produce BMP2, BMP4, and BMP6 [108]. Blocking BMP signals by the transgenic expression of Noggin results in the development of a smaller-sized mouse thymus [106].

A critical role for SMAD-mediated signaling in regulating T cell function was also reported. For instance, mice deficient in SMAD2 and SMAD3 in T cells develop early lethal autoimmunity [109,110]. In transgenic mouse models expressing SMAD7, the high-level expression of SMAD7 in T cells enhanced Th1 and Th2 cytokine production [111].

## 4. Expression and Distribution of TGF-β Family Members in the Eye

TGF-β family members exert various effects to maintain ocular homeostasis. Several members of this family, including TGF-β1, TGF-β2, TGF-β3, activin A, and inhibin, are expressed in ocular tissue [112,113] (Table 1). TGF-β 1, 2, and 3 were detected in aqueous and vitreous humors in the human eye [114]. Furthermore, these ligands are also expressed in the cornea, ciliary epithelium, crystalline lens, retina, and blood vessels [112,115,116,117,118,119]. TGF-β1 and TGF-β2 have a distinct and specific distribution in the human anterior segment of the eye, while TGF-β3 was not detected in any structure of the anterior eye [120]. TGF-β isoforms are not distributed uniformly in the human posterior eye [121]. TGF-β1 is expressed predominantly in ganglion cells, smooth muscle cells, pericytes, photoreceptors, microglia, choriocapillaris, and vitreous hyalocytes [122]. TGF-β2 is found in smooth muscle cells, pericytes, microglia, and vitreous hyalocytes, in addition to being localized in the outer segment of photoreceptors, choroidal vessels, and choroidal stroma [122,123]. TGF-β3 can be detected in choroidal histiocytes, microglia, Müller glia cells, vitreous hyalocytes, and within the mitochondria of photoreceptors [121,122]. TGF-β2 represents the preponderant isoform in the eye, since it has been found that TGF-β2 tissue concentrations were greater than TGF-β1 and TGF-β3 in vitreous, aqueous humor, and human retinal pigment epithelium (RPE) cells [124,125]. Under pathological conditions, the concentration of TGF-β2 increased in the vitreous humor of proliferative diabetic retinopathy patients and in association with the progression of retinal fibrosis. Studies from TGF-β2 knockout mice reveal ocular anomalies in the cornea, lens, and retina [126], whereas no ocular abnormalities have been reported in mice embryo lacking TGF-β1 or TGF-β3 [126]. However, Jobling et al. also demonstrated that changes in all three TGF-β isoforms expression is associated with the development of myopia [127] in an animal model.

BMP and their receptors are also expressed in adult ocular tissue as well as during the early stages of eye development. Several studies showed that BMP2, BMP3, BMP4, BMP5, BMP7, and their receptors are expressed in the adult human cornea [128,129]. BMP4 expression is more predominant in ocular tissues during development compared to other BMP members. Chang et al. showed that heterozygous deficiency of BMP4 resulted in anterior segment dysgenesis, elevated intraocular pressure, and optic nerve abnormalities similar to conditions seen in human patients with developmental glaucoma [130]. Interestingly, BMP4 may be involved in the regulation of ocular angiogenesis by promoting the secretion of VEGF by human RPE cells [131]. Mathura et al. demonstrated that BMP4 is down-regulated by injury in the retina and the RPE. These authors also determined the relative decrease in BMP4 mRNA level expression in oxygen-induced ischemia [132]. Otherwise, Shen et al. determined that BMP7 was detected in all retinal layers, with greatest expression in the inner and outer nuclear layers [133]. Three BMP receptors (BMPRIA, BMPRIB, and BMPRII) were also expressed in two anatomical structures involved in glaucoma pathogenesis, the trabecular meshwork, and optic nerve head [134].

Members of the activin/inhibin family also play significant roles in the regulation of retinal development. It has been shown that activin family members are expressed in different regions of the chick embryo retina [135]. Activin A is also involved in the eyes with proliferative vitreoretinal and seems to regulate angiogenesis and tissue fibrosis in the wound healing process [136].

**Table 1:** TGF-β expression and role in ocular pathologies.

Gene	Human/Animal Model	Ocular Pathologies	References
TGF-β1↑	In human plasma	Primary open-angle glaucoma	[137]
TGF-β1↑	In human conjunctiva and minor salivary glands	Inflammatory ocular surface [138]	
TGF-β2↑	In human aqueous humor	Proliferative vitreoretinopathy	[114]
TGF-β2↑	In human vitreous	Diabetic retinopathy	[113]
TGF-β2↑	In human aqueous humor	Open-angle glaucoma + increase of intraocular pressure in a glaucomatous eye	[139]
Activin A↑	In human vitreous specimens obtained from eyes with retinal ischemia	Regulation of angiogenesis and tissue fibrosis	[136]
BMP4↑	In adult retinal pigment epithelium-19 (ARPE-19) cells	Ocular angiogenesis associated with diabetic retinopathy via stimulation of VEGF by RPE cells	[131]
Loss of SMAD3	Human RPE-cell	Attenuation of PVR development	[126]
TGF-β1↑	In lens epithelium in mice	EMT-related fibrosis in lens epithelium	[126]
TGF-β1↑	In transgenic mice	Cataracts in the lens epithelial cells in association with EMT and accumulation of fibrous/collagenous extracellular matrix	[140]
Over-expression of TGF-β1	α-crystalline promoter in TGF-β2-null mice	Inhibition of abnormalities in ocular development (caused by the deletion of TGFβ2)	[141]
TGF-β2↓	In mouse embryo lacking TGF-β2	Loss of the corneal endothelium and anterior chamber, immature retina, and persistent vitreous vessels	[142]
Administration of anti-TGF-β2 neutralizing antibody	In mouse lens epithelium	Suppression of SMAD2/3 nuclear translocation following cataract surgery	[143]
BMP antagonist noggin	In chicken embryo lenses	Increase of cell death in lens epithelium	[144]
BMP4↓	In embryos lacking BMP4	Involvement in eye development	[145,146]
BMP4 antagonist ventroptin	In the chick eye	Alteration of several genes’ expression in the retina	[147]
Heterozygous deficiency of BMP4	In mice	Elevated intraocular pressure and optic nerve abnormalities	[130]
BMP7↓	In embryos lacking BMP7	Involvement in eye development	[145]
Adenoviral gene transfer of BMP7	In mouse lens epithelium	Suppression of injury induced EMT of lens epithelial cells and sealing of the capsular break	[148]
Targeted deletion of the BMPRIb gene	In mice	Significant elevation of apoptosis in the inner retina during postnatal development	[149]
BMPR1A↓	In lenses lacking Alk3	Abnormal lens development	[150]
TGF-βRI/RII↑	In the lens fibers of transgenic mice	Nuclear cataracts	[150]
TGF-βRII↑	In mice	Corneal opacification	[151]
Blockade of TGF-β using an adenovirus expressing an entire ectodomain of the human type II TGF-β receptor	In mice	Inhibition of the process of cornea opacification, edema and angiogenesis	[151]
SMAD3-null mice	In mouse lens epithelium with corneal exposure to alkali	Severe intraocular inflammation	[126]
Mice lacking SMAD3	In mice	Acceleration of cutaneous wound healing	
Loss of SMAD3	In mice	Blocking of morphological changes of lens epithelium and the expression of the EMT markers	[152]
Loss of SMAD3	In mice	Suppression of macrophage infiltration and growth factor expression associated to tissue destruction of the healing cornea	[152]
SMAD3 gene ablation	In mice	Attenuation of injury induced EMT of lens epithelial cells	[126]
Adenoviral gene introduction of cDNAs for SMAD7	In lens epithelium in mice	Attenuation of injury induced EMT of the lens epithelium	[153]
SMAD7 gene introduction	In mice	Attenuation of PVR development	[126]
Topical administration of SMAD7 gene introduction	In mouse cornea	Suppression of scarring and neovascularization	[126]

## 5. TGF-β in Ocular Pathologies

### 5.1. Age-Related Macular Degeneration (AMD)

AMD is a multifactorial disease involving a complex interaction between metabolic, functional, genetic variants, and environmental factors. Abnormalities associated with AMD that are not necessarily part of normal aging can be classified as neovascular or atrophic abnormalities.

*Atrophic AMD:* Drusens are the first clinical signs detectable in AMD [154]. The other lesions reported are an alteration of the pigment epithelium. RPE and the thickened inner layer of Bruch’s membrane detach and separate from the rest of Bruch’s membrane. When small, such detachment can be considered a large soft drusen. Drusens have variable effects on photoreceptors, and they can be associated with mild to moderate loss of vision. Leukocytes and macrophages have been detected in altered Bruch’s membrane and drusens [155,156]. Complement molecules have been also observed in drusens [156] as well as basal laminar deposits [157]. Investigation of isolated drusen material has shown that they elicit pro-inflammatory signals through the activation of NF-κB and the inflammasome pathways [158]. Drusens are also known to contain several factors related to inflammation, such as HLA molecules and a variety of inflammation-related proteins, such as Apolipoprotein E (ApoE), a lipophilic cholesterol transport protein, identified in hard and soft drusens [159]. The increase in size, number, and confluence of drusens leads to a risk of progression towards a choroidal neovascularization or geographic atrophy.

*Exudative AMD:* About 10% to 15% of AMD cases progress to wet AMD, which causes 80% of AMD-related severe central vision loss [160]. Neovascular AMD is characterized by proliferation of abnormal new vessels from preexisting choroidal vessels that penetrate and break through Bruch’s membrane to enter the subretinal space, which results in the disruption of the integrity of the RPE and subretinal/intraretinal hemorrhages [161,162] or even vitreous hemorrhages that could have a significant impact and devastating effect on the patient’s vision. These neovessels are accompanied by fibroblasts, producing a fibrovascular complex which can exude, bleed, and, possibly, destroy the normal architecture of the pigment epithelium–photoreceptor complex, leading to the formation of a hypertrophic fibro–glial scar. CNV may result from a local increase in angiogenic factors from the RPE/BM/choroid complex and/or a decrease in anti-angiogenic factors, leading to a disruption of the natural balance between these factors [163,164]. Indeed, the limited diffusion of growth factors between the RPE and the choriocapillaris due to lipids accumulation from the Bruch’s membrane could be an important factor in the neovascularization [165,166]. These alterations may lead to a decrease in the nutrients and the oxygen supply but can also disturb the diffusion of growth factors produced by the RPE [167].

### 5.2. TGF-β Signaling in CNV Angiogenesis

**Figure 2:**
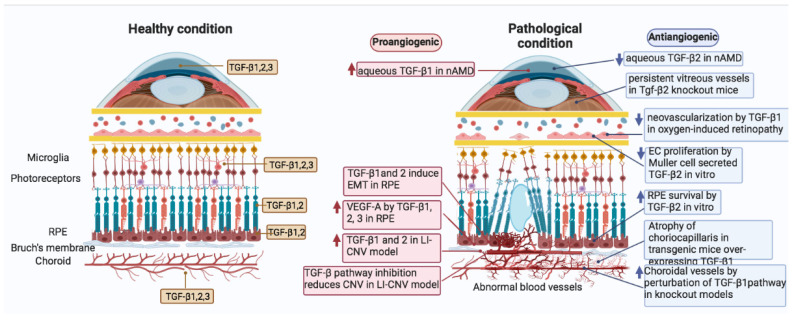
Graphic representation of the implications of TGF-β signaling in the pathological processes occurring during the progression of age-related macular degeneration. Schematic representation summarizing the TGF-β1, TGF-β2, and TGF-β3 expression in healthy condition in human eye structures and cells (**left**) and proangiogenic and antiangiogenic expression of TGF-β1, TGF-β2, and TGF-β3 in nAMD of human eye structures (**right**). The squares in blue or red indicate, respectively, down-regulation and up-regulation of different types of TGF-β with the related site in the eye. nAMD: neovascular age-related macular degeneration. EMT: epithelial to mesenchymal transition. RPE: retinal pigment epithelium. CNV: choroidal neovascularization.

Abnormal angiogenesis of the choroidal vessels is a primary cause of wet AMD, and knockout studies of TGF-β signaling showed accumulating evidence that this pathway is essential for angiogenesis. TGF-βs/BMPs have been shown to be both proangiogenic and antiangiogenic in humans (Figure 2) and in animal models linked to wet AMD [122]. Strong evidence for the proangiogenic function of TGF-β in neovascular AMD derives from the stimulation of choroidal endothelial cell proliferation and via the up-regulation of VEGF-A by RPE cells [168,169]. TGF-β1 have been extensively studied and have been implicated in angiogenesis as a pluripotent growth factor regulating cell proliferation, extracellular matrix deposition, cell migration, and macrophage infiltration, resulting in activated angiogenesis and vascular remodeling [170]. The proangiogenic function of TGF-β1 in neovascular AMD is also supported by increased concentration of total TGF-β1 found in the vitreous and aqueous humor of wet AMD patients [171,172]. At low levels, TGF-β1 leads to up-regulation of angiogenic factors while higher levels lead to the recruitment of smooth muscle cells and the inhibition of the growth of endothelial cells [173]. Both TGF-β1 and TGF-β2 are present in the region of the RPE/choroid interface [174]. TGF-β2 has been shown to have protective effects for RPE cells and physiological low levels of TGF-β2 maintain RPE homeostasis [175,176]. On the other hand, the secretion of TGF-β2 increased when human RPE cells lose polarity, which, in turn, promotes EMT, leading to RPE cells losing their normal cell shape [177], suggesting that TGF-β signaling plays different roles on RPE in the development of wet AMD.

The strongest evidence for a proangiogenic function of TGF-β subfamily members is provided by in vivo experiments of laser-induced CNV in animal models. The expression of TGF-β1 and TGF-β2 were up-regulated in choroidal and retinal cells [14] of mice subjected to laser-induced CNV [178] along with VEGF expression and SMAD2/3 phosphorylation [14]. Schlecht et al. showed that deletion of TGF-β1 and TGF-β2 signaling can induce CNV and increase the permeability of capillaries in mouse models [174]. Specific deletion of TGF-βR2 in vascular endothelial cells also induced an over-expression of VEGF-A and the development of CNV in a mouse model [174], supporting an antiangiogenic function of TGF-β family in neovascular AMD. TGF-β1, TGF-β2, and TGF-β3 induce VEGF expression in human RPE cells and, therefore, could be risk factors for the development of neovascular AMD [60]. Wang et al. also showed a significant reduction in VEGF and TNF-α protein expressions in RPE-choroid complex of mice subjected to laser-induced CNV formation after TGF-β inhibition [14].

The involvement of other TGF-β superfamily members, namely BMPs, activins, and follistatin, in angiogenesis was also highlighted in wet AMD [75,179,180,181]. Distinct BMP family members appear to be required for RPE development and have specific effects in the pathophysiology of AMD. For instance, BMP6 may act as protective factor for RPE cells from oxidative stress injury and BMP6 concentration was significantly reduced in patients with wet AMD compared with the control group [182]. On the other hand, BMP4 has been shown to be up-regulated in patients with dry AMD [183] and induces senescence in RPE cells [183]. It has been shown that BMP9 can regulate angiogenesis by blocking VEGF and basic fibroblast growth factor-induced neovascularization [184], and delivery of exogenous BMP9 has been shown to reduce CNV in mice [181]. A comprehensive analysis of in vitro experiments also showed that BMP2 stimulates VEGF release from Müller cells [185] and that Activin A is released from the mesenchyme to induce RPE development [186,187]. Similarly, Follistatin, a regulator of activin signaling, is predominantly expressed in ocular angiogenesis and was also identified as a binding partner of angiogenin, an angiogenic protein synthesized in human choroid/retina and present at high levels in AMD eyes [188].

Recently, Tosi et al. measured the protein concentration of active TGF-β1, TGF-β2, and TGF-β3 at baseline and after intravitreal anti-VEGF-A injection in the aqueous humor of patients with wet AMD: in the baseline group, the aqueous humor concentration revealed the preponderance of TGF-β2 over TGF-β3 and TGF-β1 [189]. The aqueous humor concentration revealed that TGF-β2 concentrations decreased following anti-VEGF treatments, while TGF-β1 levels remained below the dose detectable and TGF-β3 concentration was unchanged, thus suggesting a role for TGF-β2 in the homeostasis of the eye under physiological conditions and under pathological conditions in wet AMD patients [189].

### 5.3. TGF-β and Subretinal Fibrosis in Wet AMD

It is recognized that TGF-β family members are crucial cytokines in fibrogenesis [190,191], including renal fibrosis, cardiac fibrosis, and pulmonary fibrosis [192]. TGF-β family members induce a pro-fibrotic response in vitro and in vivo by increasing the expression of components of the ECM, by repressing catabolic enzymes, and by inducing the proliferation of fibroblasts as well as their differentiation into myofibroblasts. Recently, it was reported that TGF-β family members play an essential role in the formation of advanced fibrosis in CNV [193]. A rupture of immature neovessels, characteristic of wet AMD, can cause the appearance of subretinal, intra-retinal, or even vitreous hemorrhage and an exudative change leading to subretinal fibrosis, defined as the development of fibrotic material (scar tissue) which deposits and forms a sub-retinal complex due to CNV or proliferation of RPE cells.

In a mouse model [193], TGF-β1 and TGF-β2 mRNA levels were significantly elevated after subretinal fibrosis induction by subretinal injection of macrophage-rich peritoneal exudate cells, and TGF-β inhibition using a TFG-β-neutralizing antibodies (TGF-β NAb) reduced subretinal fibrosis areas by 65% compared to the control group. Recently, it was reported that TGF-β1 inhibition therapy may prevent subretinal fibrosis by inhibiting EMT [194]. Moon et al. investigated the effects of a proteasome inhibitor, bortezomib, on the EMT process in RPE cells and they showed that treatments with bortezomib block TGF-β1 signaling to decrease myofibroblastic transformation of RPE cells by down-regulating NF-κB expression. The over-expression of pro-fibrotic genes has been shown to be triggered via TGF-β1 and involves the phosphorylation of SMAD2/3 [195] or/and their nuclear accumulation, or the reduction in expression of SMAD7 signaling, thus promoting tissue fibrosis [196].

### 5.4. TGF-β Mediates Inflammatory Process Associated with Wet AMD

TGF-β family members can have pro- or anti-inflammatory effects, depending on the context. It has been shown that TGF-β1 promotes the expression of pro-inflammatory mediators, including IL-1 and IL-6, while suppressing the production of free oxygen radicals [197]. Despite its pro-inflammatory role, TGF-β1 also presents anti-inflammatory properties. In fact, targeted deletion of the mouse TGF-β1 gene leads to excessive inflammatory responses [198]. In addition, TGF-β1-null mice die from complications due to a multifocal inflammatory disorder [199]. On the other hand, TGF-β family members inhibits the immune response by stimulating regulatory T lymphocytes, blocking the proliferation of B and T lymphocytes, inhibiting the production of effector cytokines such as IL2 and IL4, blocking the function of dendritic cells, and regulating the functions of macrophages [200,201]

It is also known that TGF-β is chemotactic to macrophages leading to the induction of VEGF and monocyte chemoattractant protein 1 (MCP-1) which also have chemotactic activity [126]. Since macrophages secrete VEGF during the active phase of inflammation, the production of MCP-1 by RPE could have an influence on the development of CNV.

BMP2 can also participate in the inflammation process [185,202] and plays a pro-inflammatory role inducing leukocyte adhesion to RPE cells and the up-regulation of intercellular adhesion molecule-1 (ICAM-1), and interleukin IL-6 and 8 [185]. In the presence of BMP2, the pro-inflammatory cytokine TNF-α increased in retinal endothelial cells with no effect on the anti-inflammatory cytokines IL-4, thus contributing to pro-angiogenic and inflammatory pathways [185], suggesting that targeting BMP2 may be considered as a therapeutic approach to prevent the inflammatory pathway.

## 6. TGF-β in Other Ocular Pathologies

### 6.1. In Proliferative Vitreoretinopathy (PVR) 

PVR is the most common cause of failed repair of a primary retinal detachment [203]. Following a detachment of the retina, RPE cells, which are normally quiescent and differentiated, will detach from the Bruch’s membrane, migrate, and proliferate in the vitreous [204]. Then, epiretinal and/or vitreoretinal membranes are formed on the surface of the retina and/or in the vitreous, which will contract and pull on the retina, leading to increased detachment [204]. TGF-β family members are over-expressed in proliferative membranes in vitreoretinopathy and proliferative diabetic retinopathy (PDR) [205,206].

As previously discussed, TGF-β2 has been shown to play a role in eye homeostasis in the pathogenesis of ocular disorders, acting at the retina–RPE interface and is associated with the progression of PVR [114,115]. Contraction of hyalocyte-containing collagen gels correlates with the concentration of activated TGF-β2 in the vitreous of PDR and PVR patients [207], suggesting that TGF-β2 has a role in the formation of the proliferative membrane. Several growth factors regulate cicatricial contraction, such as PDGF and insulin-like growth factor-1 [208], and TGF-β has also been implicated also in the cicatricial contraction of the pre-retinal membranes. TGF-β family members have been especially implicated in the EMT transition of RPE and in the development of PVR, because of the profibrotic effects of TGF-β family [209]. Saika et al. also showed that SMAD3 is essential for EMT of RPE cells following retinal detachment in mice null for SMAD3 animal model, suggesting that inhibition of the SMAD3 pathway might be considered in the treatment of PVR [210]. The α-smooth muscle actin expression and myosin light chain phosphorylation were positively correlated with PDR and PVR, which were suppressed by TGF-β family blockade using a selective ROCK inhibitor and downstream mediator of TGF-β2 [207].

### 6.2. In Diabetic Retinopathy

Capillary occlusion causes retinal ischemia, due, in part, to the adhesion of macrophages to the capillary endothelium, which is a significant source of TGF-β1 in diabetic retinopathy [211]. Several studies suggest that TGF-β family members play a major role in the early pathogenesis of diabetic retinopathy (DR). Furthermore, it has been demonstrated that blockade of TGFβ-SMAD3 signaling regulates glucose tolerance and energy homeostasis, suggesting that modulating TGF-β activity may be an effective treatment strategy for obesity and diabetes [212].

BMPs are also implicated in DR. Several BMPs, including BMP4 and BMP9, have been shown to regulate glucose homeostasis and, through these effects, could have an impact on the development of diabetic retinopathy [24,213]. Recently, BMP6 has been shown to play a novel role in glucose homeostasis in type 2 diabetes mice, and treatment with BMP6 for six days resulted in improved glucose tolerance via regulation of hepatic glucose output in ob/ob mice [214]. In the eye, an elevation of BMP2 has been shown in human diabetic retina and vitreous as well as in diabetic mouse retina [185]. Increased BMP2 signaling likely contributes to DR pathophysiology by promoting VEGF release from Müller cells [185]. Similarly, BMP4-induced secretion of VEGF from RPE cells exposed to high glucose suggest that BMP4 may also play a regulatory role in angiogenesis associated with DR via VEGF synthesis and secretion [131].

A role of ALK1/BMP9 signaling has also been shown for the prevention of vascular dysfunctions in diabetic models. BMP9 levels have been shown to be decreased in patients with type II diabetes and this was associated with insulin resistance [215]. Chronic hyperglycemia impaired ALK1 signaling in endothelial cells, and Akla and colleagues reported that sustained administration of adenoviral BMP9 in diabetic mice prevented retinal vascular permeability by preventing VEGF-induced leakage and maintaining endothelial junctions [213]. BMP9/ALK1 signaling has also been shown to prevent pathological retinal neovascularization in a model of oxygen-induced retinopathy, suggesting that ALK1 agonists could be of interests for the prevention of proliferative retinopathies [181].

Finally, in DR, gremlin, a BMP2, 4, and 7 antagonist [216], has been shown to contribute to EMT in human retinal pigment epithelial cells [217]. Protein and mRNA level expression of gremlin were increased in response to high glucose in bovine retinal pericytes [218].

### 6.3. In Corneal Injury

An integrated process of cell proliferation, migration, differentiation, desquamation, and apoptosis maintain the homeostasis of adult corneal epithelium, but an alteration of these processes leads to persistent corneal anomaly, and can result in blindness. TGF-β is normally restricted to the healthy intact corneal epithelium: TGF-β1 is present within epithelial cells while TGF-β2 and β3 are detected outside epithelial cells in healthy cornea [219]. In the wounded cornea, TGF-β1 is weakly expressed while TGF-β2 is prominent. Huh et al. [220] reported differences in TGF-β3 following corneal wounding in chick cornea. TGF-βRI and II are also expressed in wounded stroma, thus participating in the wound-healing process in corneal tissue [221,222]. Following corneal injury, TGF-β family members’ over-expression leads to an increase in pro-fibrotic factors and pro-inflammatory cytokines.

BMP members also play a prominent role during the corneal wound healing response, since BMP2, 3, 4, and 5 and their receptors have been shown to be expressed in human corneal epithelium and stroma [128]. In addition, BMP7, known to be involved in the progression of EMT [223], modulates corneal wound healing through the activation of SMAD1/5/8 signaling in mouse corneal tissue [224]. In a rabbit model, the over-expression of BMP7 has been shown to modulate SMAD signaling in corneal cells [225].

Taken together, these observations support a promoting role of TGF-β family members in the course of corneal wound healing suggesting that they could represent therapeutic targets for the treatment of corneal injury.

### 6.4. In Glaucoma

Glaucoma is a progressive optic neuropathy associated with increased resistance to aqueous outflow in trabecular tissue and with elevated intraocular pressure, ocular vascular changes, and extracellular matrix remodeling. TGF-β elevation in the aqueous humor of glaucomatous eyes and the elevated intraocular pressure, trabecular meshwork (TM) obstruction, and extracellular matrix remodeling at the optic nerve head imply that dysfunctional TGF-β signaling could be involved, at least in part, in the pathology of glaucoma [226]. Indeed, it has been demonstrated that TGF-β1 and -2 induce changes in human TM cell population and phenotype contributing to increased IOP [227,228,229,230].

Numerous studies demonstrated elevated levels in the aqueous humor of patients with primary open angle glaucoma [231,232]. Biologically active levels of TGF-β2 are higher in the aqueous humor of eyes with primary open angle glaucoma than in those with primary angle closure glaucoma and uveitis-associated secondary glaucoma [233], indicating that increased levels of total and mature TGF-β2 may play an important role in the pathogenesis of primary open angle glaucoma. Using cultured trabecular meshwork cells, perfusion of TGF-β2 in anterior eye segment organ culture model caused elevated IOP and accumulation of fibrillary material in the trabecular meshwork [234].

In animal model of glaucoma, elevated levels expression of TGF-β1 and TGF-β2 were detected in the glial cells around the lamina cribrosa, suggesting that TGF-β signaling contributes to the remodeling of lamina cribrosa in glaucoma [235]. Kirwan et al. investigated the effect of exogenous TGF-β1 in human lamina cribrosa cells in vitro. The authors showed that TGF-β1 induces the expression of extracellular matrix components in glial cells of the lamina cribrosa, which may explain that the optic nerve head remodeling result, at least, from increased activation of TGF-β1 within the lamina cribrosa [236]. Using Adenoviral gene transfer to deliver active TGF-β1 to the rat eye, Robertson et al. showed anatomical changes in the anterior segment that resulted in increased intraocular pressure [230]. Bhattacharya et al. also demonstrated that TGF-β2 infusion for 14 days significantly increased intraocular pressure in cultured anterior segments [228].

BMPs have also been shown to be involved in the normal functioning of the tissues associated with the development of glaucoma, and alterations in their expression may be related to glaucoma pathogenesis. The human trabecular meshwork cells and optic nerve head express several members of the BMP family, such as BMP2, BMP4, BMP5, and BMP7. This is also the case for the receptors BMPR1a, BMPR1b, BMPR2, and the BMP antagonist gremlin. The over-expression of gremlin blocks the effect of BMP4 on TGF-β2 up-regulation of ECM deposition in the trabecular meshwork leading to elevated intraocular pressure in the perfused anterior eye organ model [134,237]. In glaucoma, elevated intraocular pressure can also be the consequence of an imbalance in the synthesis or degradation of ECM proteins, which, in turn, leads to an accumulation of ECM proteins. Wordinger et al. showed that BMP4 and BMP7 antagonize the action of TGF-β2 in human TM cells, which allows the control of ECM protein synthesis and secretion, and, thus, maintains IOP within normal values [238,239].

It has also been reported that human optic nerve head astrocytes, that are activated in glaucoma, express BMPRIA, BMPRIB, and BMPRII, which allows these cells to respond to endogenous BMP ligands [134]. This suggest that BMPs play an important role as potential signaling molecules within the optic nerve head as well as the trabecular meshwork.

Targeting TGF-β family has been proposed as a potential therapeutic measure in glaucoma. The administration of neutralizing monoclonal antibodies against human TGF-β2 (CAT-152) decreased scarring after glaucoma surgery in rabbits. Clinical trials with CAT-152 exhibited a marked improvement in patients with glaucoma undergoing surgical procedures to reduce intraocular fluid pressure [240,241]. However, no difference was shown between CAT-152 and placebo in preventing primary trabeculectomy [242,243]. Intravitreal injection of ISTH0036, an antisense oligonucleotide selectively targeting TGF-β2, indicated favorable pharmacokinetic and pharmacodynamic properties with potent and selective long tissue distribution and with down-regulation of TGF-β2 in patients with primary open angle glaucoma undergoing trabeculectomy [244].

### 6.5. In Eye Tumors

Retinoblastoma (RB) is an intraocular malignant tumor of childhood [245] that arises from pluripotent retinal progenitor cells. Mutations of the RB1 gene interfere with regulation of the cell cycle [246]. Histopathological study indicated that TGF-β1 and -2 were up-regulated in retinoblastoma tissue [247,248]. TGF-β produced by retinoblastoma cells may lead to associated cataracts in the ocular structures [247]. Although normal retinal cells have intact TGF-β signaling pathways, affinity labeling of these cells with TGF-β1 revealed that the cells did not have TGF-β receptors. This mechanism allows them to escape from negative control and form retinoblastomas [249]. The resistance to TGF-β in the human retinoblastoma cell is caused by the lack of TGF-βR-II in the retinoblastoma cells [249]. Horie et al. revealed that TGF-βR-I may be functionally inactivated in these cell lines [250]. The inhibition of ACVR1C/SMAD2 pathway using a selective inhibitor of ALK4/5/7 receptors, or down-regulation of ACVR1C or SMAD2 by shRNAs, suppressed retinoblastoma cells lines derived from primary tumors or vitreous [251,252].

Uveal melanoma is the most common form of eye cancer and the second most common site for melanoma [253]. A total of 95% of ocular melanomas are intraocular and arise from the uvea, while 5% are located in the conjunctiva. Both uveal and conjunctival melanomas are thought to derive from malignantly transformed melanocytes [254]. Myatt et al. revealed abnormalities of TGF-β in in most uveal melanoma. TGF-β1 and TGF-β2 levels are elevated in the plasma of melanoma patients, especially those with metastatic lesions, suggesting an enhanced ability of the tumor to evade host immune surveillance [255]. The eyes of human uveal melanoma are in an unusual micro-environment for immune remission. Studies have found when human uveal melanoma cell lines were treated with TGF-β, the major histocompatibility complex I expression decreased, thereby increasing the sensitivity of uveal melanoma cells to natural killer cell autolysis [256].

## 7. Therapeutic Uses of TGF-β Signaling Inhibition

With its pleiotropic activities, TGF-β family members are involved in many physiological and pathological processes. The study of the TGF-β signaling pathway has made it possible to better understand its role in various human pathologies, such as ocular diseases and tumor development. Current clinical approaches are now aimed at establishing novel cancer drugs and solid evidence for tumor suppression by TGF-β signaling was reported [257,258,259,260]. As such, there is high interest for further research and the development of new modulators of TGF-β signaling for the treatment of ocular diseases.

Currently, most therapies targeting TGF-β signaling are focused on cancer treatments or to prevent tissue fibrosis in tissues such as the heart, kidney, lungs, or liver. In cancer, therapeutic approaches targeting the signaling pathway for TGF-β family must be considered on a “case by case” since TGF-β plays contrasting roles on tumorigenesis, depending on the stage of tumor progression. Combination of vaccines and antisense oligonucleotides, and small molecules inhibitors of TGF-βR1, have shown great potential to inhibit TGF-β signaling and provide therapeutic opportunities to suppress tumor growth and metastasis [261,262]. Among the therapeutic approaches tested to inhibit the deleterious effect of TGF-β family members in fibrosis or in early stages in cancer, a natural regulator of antibodies, such as the proteoglycan decorin that can enhance or inhibit the activity of TGF-β1, or chimeric proteins TGF-βRII-IgG Fc, as well as over-expression of SMAD7 or dominant negative mutant forms of receptors, have been proposed [196,263]. Other molecules able to increase the signaling of SMADs or inhibiting the expression of SMAD7 have been proposed as chemopreventive agents. As an inhibitor of TGF-β signaling, SMAD7 is over-expressed in numerous cancer types and its abundance is positively correlated to the malignancy [264]. However, such an approach can potentially accelerate tumor development and should, therefore, be considered with caution [265,266].

### 7.1. Targeting the TGF-β Pathway for Wet AMD

**Figure 3:**
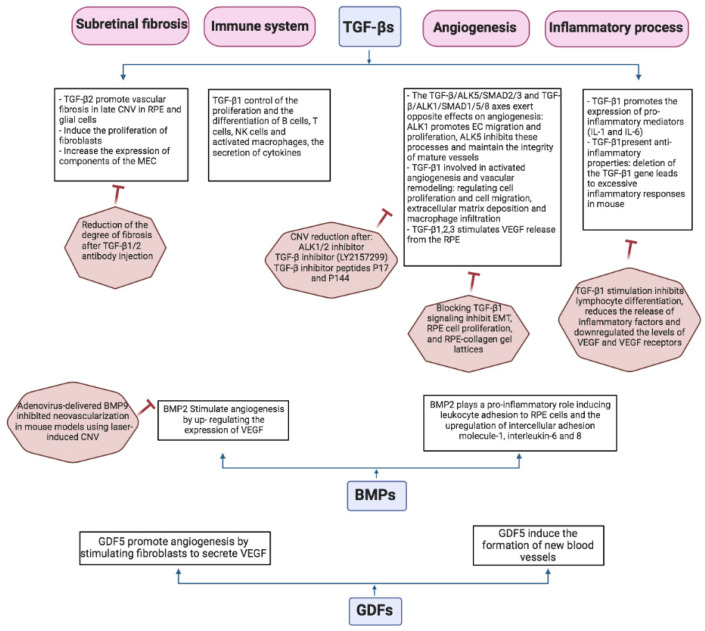
Functional implications of TGF-β signaling in pathological processes underlying age-related macular degeneration. Description of the mechanisms of TGF-β superfamily in CNV and wet AMD, including the modulation of angiogenesis-related factors, inflammation, vascular fibrosis, and immune responses. EC: endothelial cell. ECM: extracellular matrix proteins. MET: mesenchymal transition. RPE: retinal pigment epithelium. CNV: choroidal neovascularization.

Inhibition of the TGF-β pathway has been advocated as an alternative treatment for wet AMD, alongside the current treatments targeting VEGF, which prove only partially effective. TGF-β superfamily members have been revealed to participate in the development of AMD, by regulating the expression of angiogenesis-related molecules, such as VEGF, and by mediating immunoregulatory processes, inflammation, endothelial cell proliferation and migration, and the formation of fibrovascular membranes (Figure 3).

The strongest evidence for a prominent role for TGF-β signaling in wet AMD is provided by decreasing the formation of choroidal neovascularization in vivo experiments of laser-induced CNV in rodents. As discussed previously, pro- and antiangiogenic activities have been suggested for TGF-β signaling in the development of CNV, and opposite therapies have been proposed targeting the inhibition or activation of the TGF-β pathway. Using an oxygen-induced retinopathy mouse model, Kim et al. (2016) examined the possible effects of amniotic membrane-derived Mesenchymal stem cells (AMSC) on retinal neovascularization by characterizing TGF-β1 under pathological conditions and showed that intraperitoneally injected of human AMSC migrated into injured retina tissue and suppressed neovascularization through the release of ocular over-expression of TGF-β1 [267]. Zarranz-Ventura et al. demonstrated that the TGF-β inhibitor peptides P17 and P144 inhibited laser-induced CNV formation [268]. Using intraperitoneal administration of a TFG-β-neutralizing antibodies (TGF-β NAb), subretinal fibrosis was attenuated after CNV induction [193]. Given the expression of TGF-β2 in age-related macular degeneration, its therapeutic inhibition may also provide both a potent anti-fibrosis/anti-angiogenic/anti-scarring activity and a neuroprotective effect by blocking extracellular matrix remodeling. Indeed, antisense therapy targeting TGF-β2 using ISTH0036 is about to enter phase 2 clinical development for wet age-related macular degeneration and diabetic macular edema (Isarna Therapeutics).

BMPs have also been investigated as alternative targets for the prevention of ocular neovascularization. Ntumba et al. showed that adenovirus-delivered BMP9 inhibits neovascularization in laser-induced CNV in mouse [181]. In human fetal RPE cells, BMP4 was shown to inhibit TNF induced MMP-9 expression [179], which is highly expressed in CNV membranes surgically removed from AMD patients [269].

Strategies targeting TGF-β receptors have also been explored as therapeutic options for the treatment of AMD. During laser induced-CNV, inhibition of BMP signaling using LDN212854, an ALK2 specific inhibitor, suppressed CNV formation [270]. Similarly, LY2157299 (a specific TGF-β type I receptor inhibitor) or a natural TGF-β inhibitor, Decorin, significantly inhibited VEGF-A, TNF-α protein over-expression, and suppressed increased phosphorylated SMAD2/3 in retina and RPE-choroid complex of mice [14].

Otherwise, the involvement of TGF-β accessory molecules, such as LRG1 and SMOC1, have been also studied in choroidal neovascularization and could represent specific targets; SMOC1 promotes endothelial cells proliferation and angiogenesis by binding to endoglin, and LRG1 is known to promotes angiogenesis by modulating TGF-β signaling. LRG1 antibody blockade inhibits this pro-angiogenic effect by inhibiting the switch of TGF-β signaling towards ALK1 in mouse models [168,271].

### 7.2. Targeting TGF-β Signaling in Glaucoma

Many recent studies have suggested that TGF-β plays a key role in glaucoma [226,272,273]. Increased levels of TGF-β2 have been identified in the anterior chamber of glaucomatous eyes and have been linked to trabecular meshwork transformation [227]. TGF-β has also been shown to cause increased intraocular pressure and direct optic nerve damage [227]. A phase I dose escalation study investigated the safety of ISTH0036, a synthetic antisense oligodeoxynucleotide selectively targeting TGF-β2, in subjects with glaucoma undergoing trabeculectomy. The treatment aims at improving the surgery outcome by prevention of excessive scarring and trabecular meshwork transformation. After a single intravitreal administration of ISTH0036, decreased TGF-β2 mRNA and protein levels in ocular tissues were observed for up to eight weeks duration in rabbit eyes [244]. The intravitreal injection of ISTH0036 showed favorable pharmacokinetic and pharmacodynamic properties with safety results when administered in patients with primary open angle glaucoma undergoing trabeculectomy [244].

### 7.3. Targeting TGF-β Signaling in Corneal Wound Healing

TGF-β family members signaling via the SMAD pathway is probably required for the maintenance of corneal epithelial homeostasis [274]. Thus, blocking TGF-β activity at the SMAD signaling level has been suggested as a treatment option to promote corneal wound healing [274]. Indeed, blocking the activity of TGF-β family members by in vivo gene transfer of a soluble TGF-β type II receptor accelerates tissue repair in injured corneas in rats [151]. Blocking the activity of TGF-β by adenoviral gene transfer of a soluble TGF-β type II receptor results in the inhibition of corneal opacification, edema, and angiogenesis [151,275]. The use of an inhibitor to TGF-β receptor (SB431542) also maintains the normal endothelial phenotypes of cultivated corneal endothelial cells [276]. Monoclonal antibodies have also been reported to be potential treatments for corneal scarring: TGF-β antagonists, such as TGF-β1 and β2 antibodies, have been shown to inhibit the formation of cutaneous scars in rodent wounds [277]. The use of Tranilast, a TGF-β inhibitor, reduced the re-occurrence of corneal fibrosis, or primary pterygium, a degenerative ocular surface disorder with fibrovascular growth of the bulbar conjunctiva onto the cornea [278].

TGF-β/SMAD signaling is believed to play a role in wound healing of corneas affected by Stevens–Johnson’s syndrome or chemical burns [274,279]. TGF-β isoforms have been detected in corneal epithelium and are also expressed in wounded stroma, suggesting their participation in the wound-healing process in corneal tissue [126]. The inhibition of TGF-β signaling via the over-expression of regulatory SMAD7 following corneal injury has also been shown to be associated with scar formation. Tissue repair has been observed in damaged corneas after blocking TGF-β activity by systemic expression of the soluble TGF-β receptor by transfer of adenoviral genes [151].

Stevens–Johnson’s syndrome is another inflammatory corneal disease caused by an autoimmune mechanism. The levels of TGF-β1 were quantitated by enzyme-linked immunosorbent assay method and compared between Stevens–Johnson’s syndrome cases and age-matched/sex-matched healthy controls. Elevated tear TGF-β1 profiling of patients with Stevens–Johnson’s suggests that TGF-β1 plays an inflammatory role in the pathogenesis [280]. Thus, it would seem that interference of TGF-β signaling might have a therapeutic effect on this disorder.

## 8. Conclusions

It is increasingly apparent that TGF-β family members play a prominent role in the regulation of cell behavior in ocular tissues in physiological and pathological processes of development or tissue repair. Emerging evidence has shown that therapeutic strategies based on the modulation of TGF-β in AMD could constitute a promising avenue to treat this major cause of blindness by regulating several pathophysiological processes involved in the disease, including fibrosis, the immune response, and RPE function as well as the angiogenic response. Indeed, several molecules involved in the modulation of this pathway that are currently investigated in pre-clinical or clinical studies have shown promising potential for the treatment of the disease. Further understanding of the regulation of TGF-β in CNV processes will be needed to develop new strategies for the treatment of wet AMD and other ocular pathologies.
